# Multilayer 3D Chiral Folding Polymers and Their Asymmetric Catalytic Assembly

**DOI:** 10.34133/2022/9847949

**Published:** 2022-02-16

**Authors:** Yao Tang, Shengzhou Jin, Sai Zhang, Guan-Zhao Wu, Jia-Yin Wang, Ting Xu, Yu Wang, Daniel Unruh, Kazimierz Surowiec, Yanzhang Ma, Shiren Wang, Courtney Katz, Hongjun Liang, Yunze Li, Weilong Cong, Guigen Li

**Affiliations:** ^1^Department of Chemistry and Biochemistry, Texas Tech University, Lubbock, Texas 79409-1061, USA; ^2^Institute of Chemistry and Biomedical Sciences, School of Chemistry and Chemical Engineering, Nanjing University, Nanjing 210093, China; ^3^Department of Mechanical Engineering, Texas Tech University, Lubbock, Texas 79409-1061, USA; ^4^Department of Industrial & Systems Engineering, Texas A&M University, College Station, Texas 77843, USA; ^5^Department of Cell Physiology and Molecular Biophysics, School of Medicine, Texas Tech University Health Sciences Center, Lubbock, TX 79430-6551, USA; ^6^Department of Industrial Engineering, Texas Tech University, Lubbock, Texas 79409-3061, USA

## Abstract

A novel class of polymers and oligomers of chiral folding chirality has been designed and synthesized, showing structurally compacted triple-column/multiple-layer frameworks. Both uniformed and differentiated aromatic chromophoric units were successfully constructed between naphthyl piers of this framework. Screening monomers, catalysts, and catalytic systems led to the success of asymmetric catalytic Suzuki-Miyaura polycouplings. Enantio- and diastereochemistry were unambiguously determined by X-ray structural analysis and concurrently by comparison with a similar asymmetric induction by the same catalyst in the asymmetric synthesis of a chiral three-layered product. The resulting chiral polymers exhibit intense fluorescence activity in a solid form and solution under specific wavelength irradiation.

## 1. Introduction

The search for desired and challenging properties of materials heavily depends on the molecular design and synthesis of monomers and polymers [1–10]. The discovery and development of new polymers, especially conductive polymers, have become one of the most active topics in modern materials science in the past several decades [11–17]. The properties of conductive polymers are mainly attributed to the electronic flexibility conjugated through their carbon-carbon double and triple bonds of the backbones of their frameworks [18–20]. In addition to electronic delocalization, through-space conjugation has emerged as an alternative pathway for energy and charge transfers in polymers [16, 17, 21–23]. For example, a five-layered congener of decked naphthalene-diimides (NDIs) anchored by 1,8-diethynylanthracene spacers displayed a ten-electron reversible reduction process in a small working potential window (~0.8 V), which is abnormal electronic behavior in organic conductive frameworks. Meanwhile, multiple-layered polymers, such as poly-(dibenzofulvene)s, [2.2]paracyclophane-layered, and 2D multilayered *π*-stacked conjugated polymers, have been established and displayed various attractive properties [24].

**Figure 1:**
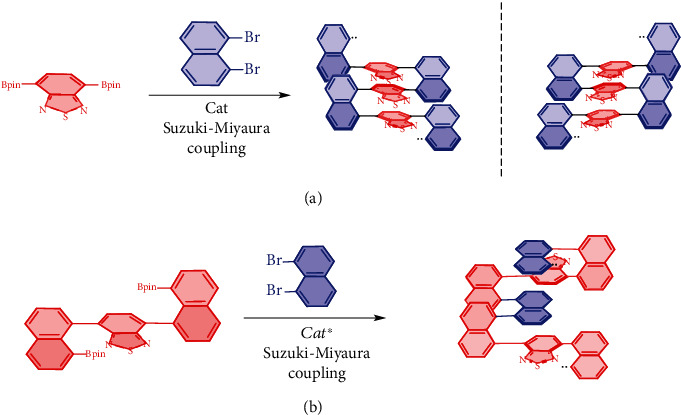
(a) Polymerization of racemic polymers. (b) Asymmetric catalytic polymerization.

Recently, our lab has designed and synthesized novel multilayer 3D racemic polymers and corresponding oligomers via the Suzuki-Miyaura catalytic coupling (Figure 1(a)). In these polymers, nearly parallel uniformed aromatic bridges (red segment, Figure 1(a)) exist between two columns of naphthalene-holding skeletons. X-ray analysis and computational studies have unambiguously confirmed the structurally condensed and regularly stacked patterns. These polymers displayed strong luminescence in the solid-state under UV irradiation and photoluminescence (PL). So far, the work on enantiomerically enriched polymers tightly compacted with folded-stacking has been left behind. In fact, an asymmetric catalytic approach to multiple-columned and multiple-layered polymers has not appeared in literature, yet this is probably due to extreme difficulties in finding suitable catalysts and conditions for the synthesis. However, the research on chiral polymers has become increasingly important as more potential applications in materials [11–17], such as chiral switches, chemical and biological sensors/probes, and liquid crystals for three-dimensional displays, circularly polarized luminescence (CPL) to complement, or even to replace, classical luminescent materials, etc.

In this letter, we would like to report our preliminary results on asymmetric catalytic polymerization accessing novel triple-columned and multiple-layered chiral polymers by searching for efficient chiral catalytic systems under multiple Suzuki-Miyaura cross-couplings (Figure 1(b)).

## 2. Results

In our previous design of multiple-layered 3D polymers, the bridges between column anchors were either symmetrically or nonsymmetrically substituted aromatic rings on their 1,4-position [25]. In the present design, the derivatives of 1,8-dibromonaphthalene were employed as building blocks alternatively together with benzo[c] [1, 2, 5], thiadiazole and benzo[c] [1, 2, 5], and selenodizole scaffolds [26]. The resulting polymers in this work displayed different arrangements by layered-column anchors in which a long-distance existed between each pair of anchor planes than that of previous ones. This phenomenon was due to an extra naphthalene ring inserted between each pair of anchor planes.

**Figure 2:**
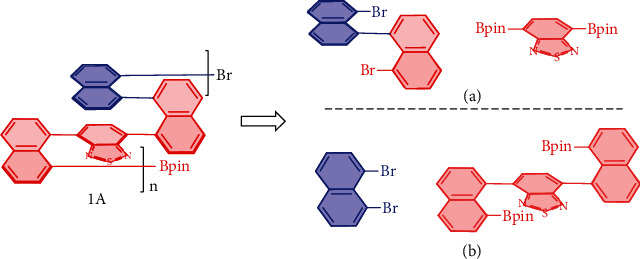
Retrosynthetic analysis of chiral polymers.

The retrosynthetic analysis (RSA) [27] was conducted by using 1A as a representative through disconnections into two pairs of synthons (Figures 2(a) and 2(b)). The purpose of selecting thiadiazole synthon for this polymerization was based on the fact that it had been among the most frequently employed scaffolds in polymers and materials science, especially in the field of conductive polymers. The synthesis of the monomer of 8,8′-dibromo-1,1′-binaphthalene was very challenging since one of the critical steps involved the cross-coupling between 2-(8-bromonaphthalen-1-yl)-4,4,5,5-tetramethyl-1,3,2-dioxaborolane and 1-bromo-8-iodonaphthalene taking about two weeks in a poor yield [28]. Therefore, the first synthon pair was excluded temporarily. We, therefore, chose the second synthon pair for the present asymmetric catalytic polymerization.

**Scheme 1:**
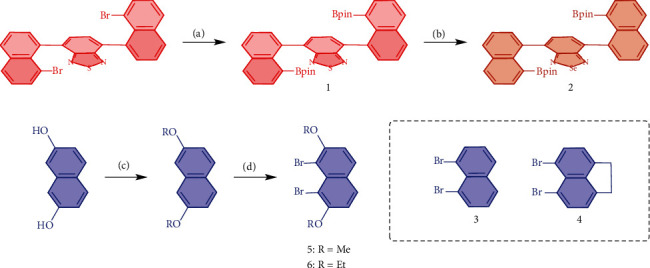
Monomer synthesis. Conditions: (a) Pd(dppf)Cl_2_, 1,4-dioxane, 108°C, 12 h, 65%; (b) NaBH_4_, CoCl_2_∙6H_2_O, THF/EtOH, r.t., 4 h; SeO_2_, EtOH/H_2_O, reflux, 8 h; (c) MeI, Acetone, 0°C/EtBr, DMF, 70°C; and (d) NBS, pyridine, CHCl_3_, reflux.

The comonomer 1 was synthesized starting from 4,7-bis(8-bromonaphthalen-1-yl)benzo[c][1,2,5]thiadizole [26, 29] through the Miyaura borylation reaction in a yield of 65%. Selenodiazole derivative two served as an additional basic skeleton which was achieved by following the reported procedure with 47% yield (Scheme 1) [19, 23]. Methylation/ethylation of naphthalene-1,8-diol, followed by bromination with NBS in the presence of pyridine, afforded the target monomers 5 and 6 with a yield of 60% and 75%, respectively. The comonomers (3 and 4) were purchased from commercial sources and directly employed for the polymerization without further purification.

Taking 1A as the target, we screened a series of mono- and bisphosphines and Pd-ligand complexes to get the desired polymer. Most of the examined chiral catalysts were proven to be ineffective, giving either no polymer products observed or complex mixtures with no optical rotation, except for Pd(S-BINAP)Cl_2_, which provided the best result with a chemical yield of 65% and optical rotation of [*α*]_D_^20^ = −7.1.

**Scheme 2:**
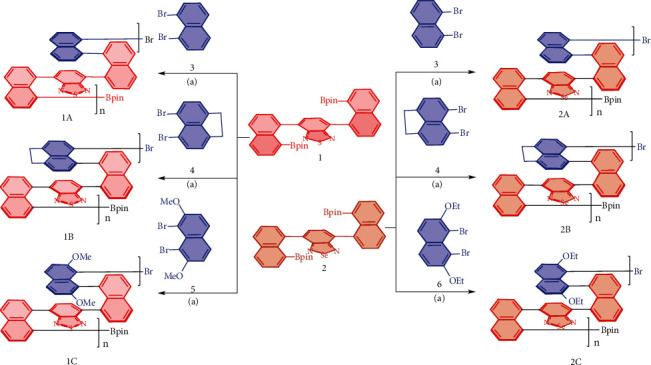
The catalytic coupling assembly of multilayer 3D polymers 1A-2C; conditions: Pd(S-BINAP) Cl_2_, THF/H_2_O, K_2_CO_3_, 85°C, 6 days.

As shown in Scheme 2, six pairs of comonomers were investigated, showing a good substrate scope, albeit many other similar substrates had not been examined. While S- and Se-containing bridges were electron-deficient aromatics in the middle of column anchors, their surrounding layers varied from neutral aromatic ring (naphthalene moiety) to electron-enriched moieties with two MeO-/EtO- groups. These differentiated-layer arrangements would benefit the search for challenging properties of materials, such as optoelectronics, photovoltaics, and polarized organic electronics, etc.

**Figure 3:**
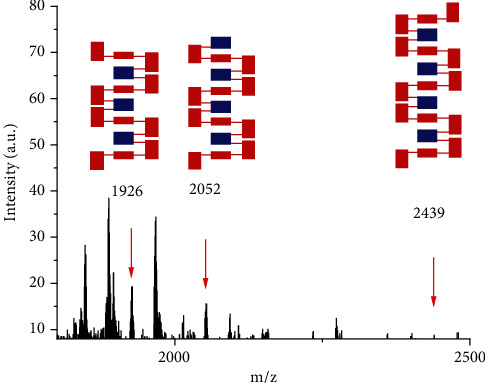
MALDI-TOF of chiral oligomer 1A.

The synthetic and analytical results of six types of chiral polymers are summarized in Table 1. At the same time, 1A and 1C showed extremely bad solubility in THF, which made gel permeation chromatography (GPC) analysis difficult. The other four polymers enabled GPC analysis to be conducted smoothly and showed *M*_w_ arranging from 53,136 to 72,321, and *M*_n_ from 40,854 to 51,135, respectively (Figure. S13-S16). For cases 1A and 1C, the MALDI-TOF analysis showed the highest molecular weights (*M*_w_) as 2439 and 1533, indicating the existence of nine and five layers in the structures, respectively (Figure 3 and S17).

**Figure 4:**
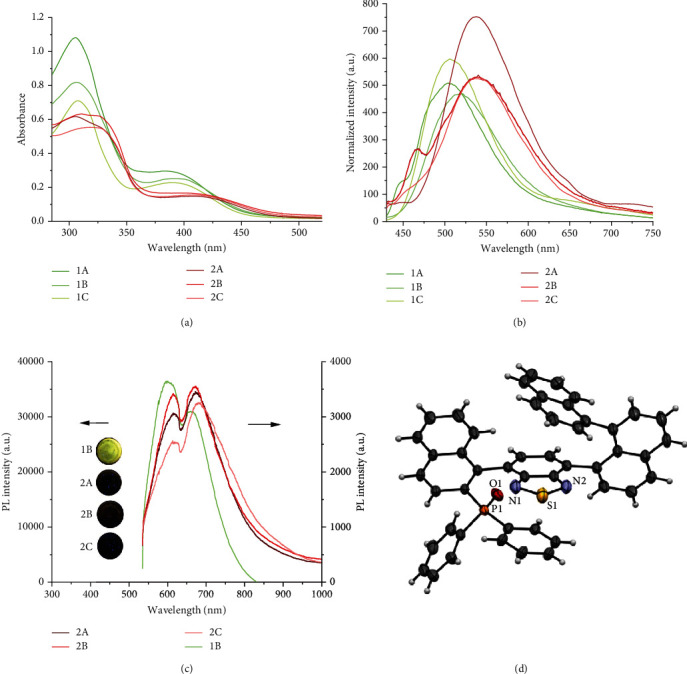
(a) UV-Vis absorbance of 1A-1C and 2A-2C (0.05 mg/mL) in CHCl_3_. (b) Normalized fluorescent spectra of 1A-2C (0.05 mg/mL) in THF. Excitation wavelength for 1A-1C: 390 nm; excitation wavelength for 2A: 310 nm; excitation wavelength for 2B and 2C: 410 nm. (c) Photoluminescence (PL) spectra of solid samples 1B, 2A-2C; excitation wavelength (*λ*ex): 532 nm. Inset: colors of solid samples 1B and 2A-2C under 365 nm UV lamp. (d) Diastereochemistry assignment of a comonomer.

Our attempt to obtain pure oligomers to conduct X-ray structural analysis had not been successful due to too many compounds coexisting in the resulting mixtures; this made purification extremely difficult. However, major enantiomers' absolute structure could be compared with a similar enantioselective induction by the same catalyst of Pd(*S*-BINAP)Cl_2_. This catalyst generated “*S*”-shaped multilayer 3D enantiomers compared with the orientation of heterocyclic ring on the bridge in our previous work and was anticipated to give the same asymmetric induction in this polymerization as shown in Figure 4(d) [30]. Interestingly, this catalyst led to a new chiral framework containing a pseudocenter chirality and orientational/rotational chirality. The The pseudo or pro-chirality center is generated at a phosphorus atom with two identical aryl rings but differentiated by aromatic packing and unpacking, respectively. This pseudo or pro-chiral center can be extended to other tetrahedron or polyhedron centers (e.g., C, Si, etc.) including those centers attached by four different groups. The orientation/rotational chirality is realized by atropisomeric rotation along axis of the P-C bond which is reinforced by naphthalenyl ring and three moieties of diarylphosphine oxide scaffolds (two differentiated aromatic rings and one P=O group).

The diastereochemistry of bridge arrangements of polymeric products could be readily assigned by X-ray structural analysis of a Suzuki-Miyaura cross-coupling product controlled by the same catalyst of Pd(*S*-BINAP)Cl_2_ as well (Figure 4(d)). In this parallel structural arrangement, steric effects between side rings of naphthalene (bridge) and [1,2,5]thiadiazole could direct the orientation of their diastereochemistry. In fact, it was the first time for us to obtain this single crystal of asymmetric catalytic Suzuki-Miyaura cross-couplings participated by two nonsymmetric wings during controlling layered structures.

The UV-Vis absorptions of 1A-1C and 2A-2C in CHCl_3_ are displayed in Figure 4(a). 1A-1C exhibited the maximum absorptions at 305 nm and broad absorption between 365 nm and 430 nm. Compared with benzothiadiazole polymers, both peaks of the benzoselenadiazole polymers 2A-2C red-shifted slightly and exhibited two broad absorptions in the range of 285 to 345 nm and 370 to 470 nm.

The fluorescence studies of 1A-2C were performed in THF at room temperature. In Figure 4(b), 1A-1C emitted yellow-green fluorescence with maximum emission peaks around 510 nm. Obviously, replacing benzothiadiazole foldamers with benzoselenadiazole counterparts made the maximum fluorescent emission peaks shift toward a long wavelength to 540 nm (2A-2C).

In order to gain an insight into the solid emission, four selected solid samples were carried out the solid-state photoluminescence (PL) spectra (Figure 4(c)) upon excitation at 532 nm. Notably, solid 1B displayed much stronger PL intensity (10 times) than the other three samples (2A-2C), which was consistent with the observation in the inset photo. In addition, all samples exhibited two peaks at around 610 nm and 670 nm, in which only 1B exhibited a more substantial emission peak around 610 nm, while the higher intensities of 2A-2C were shown around 670 nm. Under a 365 nm UV lamp, the luminescence of four solid samples (1B, 2A-2C) were examined, whereas just 1B displayed a light yellow emission under 365 nm excitation while the emissions of 2A-2C were not observable (Figure 4(c)).

**Figure 5:**
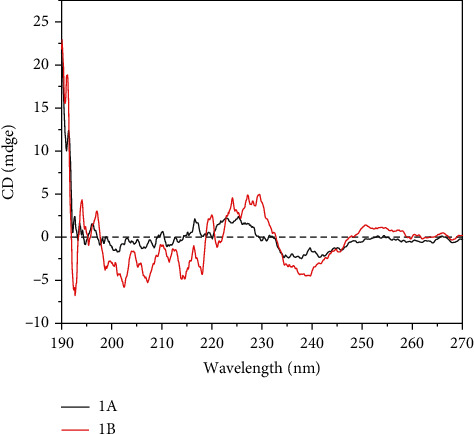
CD spectra of 1A and 1B in methanol; *c* = 0.2 mg/mL.

The optical activities of selected chiral polymers 1A and 1B were further studied by CD spectroscopies in methanol. The only optical absorption appeared between 190 nm and 260 nm due to the *π* − *π*^∗^ transition of aromatic rings. As exhibited in Figure 5, polymer 1A showed negative Cotton effects in the range of 190-195 nm, 197-208 nm, 210-211 nm, 217-220 nm, and 225-238 nm, while it showed positive Cotton effects in the range of 195-197 nm, 208-210 nm, 211-217 nm, 220-225 nm, and >238 nm. A similar chiral environment of the polymer backbone can be observed on polymer 1B either, featuring negative Cotton effects centered at about 192 nm and 240 nm, and gradually turned to the positive Cotton effect, respectively. The Cotton effects from polymers 1C, 2A, 2B, and 2C were less intense than 1A and 2B (Figure S19). The information from the Cotton effects indicates that all chiral polymer products have been controlled consistently in regard to their absolute stereochemistry.

**Figure 6:**
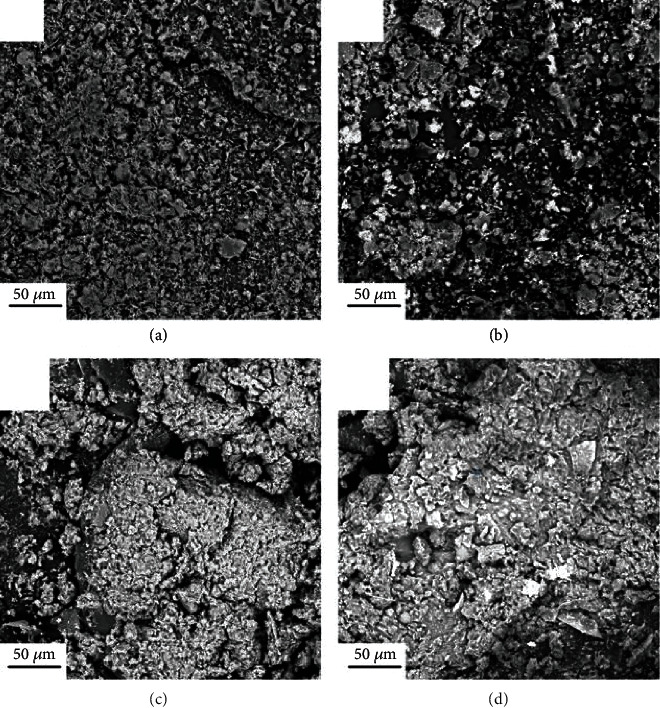
SEM images of chiral folding polymers (a) 1A, (b) 1B, (c) 2A, and (d) 2B.

Morphological studies of chiral polymers 1A, 1B, 2A, and 2B were performed by scanning electron microscopy (SEM). A thin gold layer was applied to coat all the polymer samples to increase their conductivity and decrease the signal-to-noise ratio.  Figure 6(a) (polymer 1A) and Figure 6(b) (polymer 1B) revealed some homogeneous balls with lengths around 5 *μ*m in diameters dispersedly tangled into a porous and flat mat. On the other hand, polymer 2A (Figure 6(c)) and polymer 2B (Figure 6(d)) exhibited a more dense and compact texture than polymers 1A and 1B. The sizes of the gray basement in compounds shown in Figures 6(c) and 6(d) are much larger than those shown in Figures 6(a) and 6(b). In addition, different from the compounds shown in Figures 6(a) and 6(b), some semblable cauliflower-like surface structures can be found in the compounds shown in Figures 6(c) and 6(d).

## 3. Discussion

In summary, we have designed and synthesized a novel class of structurally compacted triple-column/multiple-layer chiral 3D folding polymers and oligomers. The resulting chiral polymers contain both uniformed and differentiated aromatic bridges in the middle of two bridge columns, with their structures determined by the GPC and MALDI-TOF analyses. Corresponding multiple-layered chiral oligomers were proven to exist during the polymerization process, as shown in MALDI-TOF spectra. Absolute stereochemistry (enantio- and diastereochemistry) was assigned by X-ray structural analysis of their monomers and, concurrently, by comparison with a similar asymmetric induction by the same catalyst in our previous asymmetric synthesis of chiral three-layered products. Chiral polymers in this work exhibit fluorescence activity in solid form and solution under specific wavelength irradiation. A series of derivatives based on present chiral polymers will be designed and synthesized, achieving challenging properties in our labs.

## Figures and Tables

**Table 1:** Results of synthetic polymers.

Entry	Product	*M*_n_^[a]^	*M*_w_^[a]^	PDI^[a][b]^	MALDI-TOF^[c]^	[*α*]_D_^RT^^[d]^
1	1A	—	—	—	2439	-7.1 (*c* = 0.14)
2	1B	51,135	72,321	1.41	—	+6.4 (*c* = 0.14)
3	1C	—	—	—	1533	+5.9 (*c* = 0.19)
4	2A	42,126	58,570	1.39	—	+4.6 (*c* = 0.15)
5	2B	45,143	55,199	1.22	—	+11 (*c* = 0.08)
6	2C	40,854	53,136	1.30	—	+15 (*c* = 0.08)

^[a]^Determined by GPC with a polystyrene standard. ^[b]^PDI = *M*_w_/*M*_n_. ^[c]^Based on the analysis of crude reaction mixture. ^[d]^Calculated by using clear solutions in chloroform.

## Supplementary Materials

Supplementary Materials Figure S1: ^1^H NMR spectrum of 1,8-dibromo-2,7-diethoxynaphthalene. Figure S2: ^13^C NMR spectrum of 1,8-dibromo-2,7-diethoxynaphthalene. Figure S3: ^1^H NMR spectrum of 1. Figure S4: ^13^C NMR spectrum of 1. Figure S5: ^1^H NMR spectrum of 2. Figure S6: ^13^C NMR spectrum of 2. Figure S7: ^1^H NMR spectrum of 1A. Figure S8: ^1^H NMR spectrum of 1B. Figure S9: ^1^H NMR spectrum of 1C. Figure S10: ^1^H NMR spectrum of 2A. Figure S11: ^1^H NMR spectrum of 2B. Figure S12: ^1^H NMR spectrum of 2C. Figure S13: GPC data of 1B. The inset shows the zoomed in rectangular area. Figure S14: GPC data of 2A. The inset shows the zoomed in rectangular area. Figure S15: GPC data of 2B. The inset shows the zoomed in rectangular area. Figure S16: GPC data of 2C. The inset shows the zoomed in rectangular area. Figure S17: MALDI-TOF data of 1C. Figure S18: MALDI-TOF data of 1A. Table S1: Crystal data and structure refinement for comonomer. Figure S19: CD spectra.

## References

[1] Skotheim T. A., Elsenbaumer R. L., Reynolds J. R. (1998). *Handbook of conducting polymers*.

[2] Thomas S. W., Joly G. D., Swager T. M. (2007). Chemical sensors based on amplifying fluorescent conjugated polymers. *Chemical Reviews*.

[3] Gellman S. H. (1998). Foldamers: a manifesto. *Accounts of Chemical Research*.

[4] Tour J. M. (2000). Molecular electronics. Synthesis and testing of components. *Accounts of Chemical Research*.

[5] Tour J. M. (1996). Conjugated macromolecules of precise length and constitution. Organic synthesis for the construction of nanoarchitectures. *Chemical Reviews*.

[6] Xu L., Wang C., Li Y.-X. (2020). Crystallization-driven asymmetric helical assembly of conjugated block copolymers and the aggregation induced white-light emission and circularly polarized luminescence. *Angewandte Chemie, International Edition*.

[7] Liu W. B., Xu X. H., Kang S. M. (2021). Bottlebrush polymers carrying side chains on every backbone atom: controlled synthesis, polymerization-induced emission, and circularly polarized luminescence. *Macromolecules*.

[8] Liu N., Zhou L., Wu Z.-Q. (2021). Alkyne-palladium(II)-catalyzed living polymerization of isocyanides: an exploration of diverse structures and functions. *Accounts of Chemical Research*.

[9] Zhou L., Xu X. H., Jiang Z. Q. (2021). Selective synthesis of single-handed helical polymers from achiral monomer and a mechanism study on helix-sense-selective polymerization. *Angewandte Chemie, International Edition*.

[10] Chu J.-H., Xu X.-H., Kang S.-M., Liu N., Wu Z.-Q. (2018). Fast living polymerization and helix-sense-selective polymerization of diazoacetates using air-stable palladium(II) catalysts. *Journal of the American Chemical Society*.

[11] Gutzler R., Perepichka D. F. (2013). *π*-Electron conjugation in two dimensions. *Journal of the American Chemical Society*.

[12] Messier J., Prasad P., Ulrich D. (2012). *Nonlinear optical effects in organic polymers*.

[13] Li J., Shen P., Zhao Z., Tang B. Z. (2019). Through-space conjugation: a thriving alternative for optoelectronic Materials. *Chemistry*.

[14] Wang X., Lou X. Y., Jin X. Y., Liang F., Yang Y. W. (2019). A binary supramolecular assembly with intense fluorescence emission, high pH stability, and cation selectivity: supramolecular assembly-induced emission materials. *Research*.

[15] Xu H., Liu L., Teng F., Lu N. (2019). Emission enhancement of fluorescent molecules by antireflective arrays. *Research*.

[16] Niu G., Zheng X., Zhao Z. (2019). Functionalized acrylonitriles with aggregation-induced emission: structure tuning by simple reaction-condition variation, efficient red emission, and two-photon bioimaging. *Journal of the American Chemical Society*.

[17] Xu L., Wang Z., Wang R. (2020). A conjugated polymeric supramolecular network with aggregation-induced emission enhancement: an efficient light-harvesting system with an ultrahigh antenna effect. *Angewandte Chemie, International Edition*.

[18] Hastings J., Pouget J. P., Shirane G., Heeger A. J., Miro N. D., MacDiarmid A. G. (1977). one-dimensional phonons and "Phase-Ordering" phase transition _inHg3−*δ*AsF6_. *Physical Review Letters*.

[19] Shirakawa H., Louis E. J., MacDiarmid A. G., Chiang C. K., Heeger A. J. (1977). Synthesis of electrically conducting organic polymers: halogen derivatives of polyacetylene, (CH)x. *Journal of the Chemical Society, Chemical Communications*.

[20] Shirakawa H., McDiarmid A., Heeger A. (2003). Focus Article: Twenty-five years of conducting polymers. *Chemical Communications*.

[21] Nakano T. (2010). Synthesis, structure and function of *π*-stacked polymers. *Polymer Journal*.

[22] Mukhopadhyay S., Jagtap S. P., Coropceanu V., Brédas J. L., Collard D. M. (2012). *π*-Stacked oligo (phenylene vinylene) s based on pseudo-geminal substituted [2.2] paracyclophanes: impact of interchain geometry and interactions on the electronic properties. *Angewandte Chemie, International Edition*.

[23] Spuling E., Sharma N., Samuel I. D., Zysman-Colman E., Bräse S. (2018). (Deep) blue through-space conjugated TADF emitters based on [2.2] paracyclophanes. *Chemical Communications*.

[24] Keshri S. K., Ishizuka T., Kojima T., Matsushita Y., Takeuchi M. (2021). Long-range order in supramolecular *π* assemblies in discrete multidecker naphthalenediimides. *Journal of the American Chemical Society*.

[25] Wu G., Liu Y., Yang Z. (2021). triple-columned and multiple-layered 3D polymers: design, synthesis, aggregation-induced emission (AIE), and computational study. *Research*.

[26] Neto B. A. D., Lopes A. S., Wüst M., Costa V. E. U., Ebeling G., Dupont J. (2005). Reductive sulfur extrusion reaction of 2,1,3-benzothiadiazole compounds: a new methodology using NaBH_4_/CoCl_2_∗6H_2_O_(cat)_ as the reducing system. *Tetrahedron Letters*.

[27] Corey E. J. (1995). *The logic of chemical synthesis*.

[28] Regulska E., Ruppert H., Rominger F., Romero-Nieto C. (2020). Synthesis of blue-luminescent seven-membered phosphorus heterocycles. *The Journal of Organic Chemistry*.

[29] Wu G., Liu Y., Yang Z. (2020). Enantioselective assembly of multi-layer3Dchirality. *National Science Review*.

[30] Wu G., Liu Y., Rouh H. (2021). Asymmetric catalytic approach to multilayer 3D chirality. *Chemistry - A European Journal*.

