# Supplementary material for: Multilayer 3D Chiral Folding Polymers and Their Asymmetric Catalytic Assembly
Source: Research (Wash D C). 2022 Feb 16;2022:9847949. doi: 10.34133/2022/9847949 (PMC8873952; doi:10.34133/2022/9847949)
Supplement: Supplementary Materials — Figure S1: 1H NMR spectrum of 1,8-dibromo-2,7-diethoxynaphthalene. Figure S2: 13C NMR spectrum of 1,8-dibromo-2,7-diethoxynaphthalene. Figure S3: 1H NMR spectrum of 1. Figure S4: 13C NMR spectrum of 1. Figure S5: 1H NMR spectrum of 2. Figure S6: 13C NMR spectrum of 2. Figure S7: 1H NMR spectrum of 1A. Figure S8: 1H NMR spectrum of 1B. Figure S9: 1H NMR spectrum of 1C. Figure S10: 1H NMR spectrum of 2A. Figure S11: 1H NMR spectrum of 2B. Figure S12: 1H NMR spectrum of 2C. Figure S13: GPC data of 1B. The inset shows the zoomed in rectangular area. Figure S14: GPC data of 2A. The inset shows the zoomed in rectangular area. Figure S15: GPC data of 2B. The inset shows the zoomed in rectangular area. Figure S16: GPC data of 2C. The inset shows the zoomed in rectangular area. Figure S17: MALDI-TOF data of 1C. Figure S18: MALDI-TOF data of 1A. Table S1: Crystal data and structure refinement for comonomer. Figure S19: CD spectra. [file 9847949.f1.pdf]

Supporting Information for

Multi-Layer 3D Chiral Folding Polymers and Their Asymmetric Catalytic Assembly

Yao Tang,[1,†] Shengzhou Jin,[2,†] Sai Zhang,[1,†] Guan-Zhao Wu,[1] Jia-Yin Wang,[2] Ting Xu,[2] Yu Wang,[2] Daniel Unruh,[1] Kazimierz Surowiec,[1] Yanzhang Ma,[3] Shiren Wang,[4] Courtney Katz,[5] Hongjun Liang,[5] Yunze Li,[6] Weilong Cong,[6] and Guigen Li[1,2,*]

[1]*Department of Chemistry and Biochemistry, Texas Tech University, Lubbock, Texas 79409-1061, USA.*

[2]*Institute of Chemistry and Biomedical Sciences, School of Chemistry and Chemical Engineering, Nanjing University, Nanjing, 210093, China.*

[3] *Department of Mechanical Engineering, Texas Tech University, Lubbock, Texas 79409-1061, USA.*

[4] *Department of Industrial & Systems Engineering, Texas A&M University, College Station, Texas 77843, USA.*

[5] *Department of Cell Physiology and Molecular Biophysics, School of Medicine, Texas Tech University Health Sciences Center, Lubbock, TX 79430-6551.*

[6]*Department of Industrial Engineering, Texas Tech University, Lubbock, Texas 79409-3061.*

[*]Correspondence should be addressed to Guigen Li; guigen.li@ttu.edu.

[†]These authors contributed equally to this work.

Table of Contents

1. **General Information**……………………………………………………S2
2. **Synthetic Procedures**………………………………………………....S4
3. **NMR Spectra**………………………………………………………… S8
4. **GPC Data**……………………………………………………………….S14
5. **MALDI-TOF Analysis**…………………………………………………..S16
6. **X-ray Diffraction Information** ………………………………………..S17
7. **CD Spectra**……………………………………………………………..S19

1. General Information

Unless otherwise stated, all reactions were magnetically stirred and conducted in oven-dried glassware in anhydrous solvents under Ar. Heated oil baths were used for reactions requiring elevated temperatures. Solvents were removed under reduced pressure at 40-65 °C using a rotavapor. All given yields are isolated yields of chromatographically and NMR spectroscopically materials.

All commercially available chemicals were used as received without further purification. Solvents as follows: MeOH, EtOH, toluene, DMF, EtOAc, DCM, hexane, THF, acetone and 1,4-dioxane were used without further purification.

The ^{1}H and ^{13}C NMR spectra were recorded in $CDCl_3$ on 400 MHz and 100 MHz instruments with TMS as internal standard. For referencing of the ^{1}H NMR spectra, the residual solvent signal (δ = 7.26 for $CDCl_3$) was used. In the case of the ^{13}C NMR spectra, the signal of solvents (δ = 77.06 ± 0.03 for $CDCl_3$ and δ = 39.52 for DMSO-d_6) were reported in ppm with respect to TMS. Data are represented as follows: chemical shift, multiplicity (s = singlet, d = doublet, t = triplet, m = multiplet), coupling constant (*J*, Hz), and integration. MALDI-TOF analyses were carried out using an ABI/MDS SCIEX 4800 Mass Spectrometer with HABA matrix. UV-Vis spectra were collected on an Agilent 8453 UV-Visible Spectroscopy system. Fluorescence data were collected by using cary Eclipse Fluorescence Spectrophotometer and Eclipse ADL program.

GPC data were collected by using TOSOH EcoSEC HLC-8320 GPC equipped with a dual-flow refractive index detector. A UV detector is also included for UV visible polymers and can be used in tandem with the RI detector. The installed columns have a range of 500-10^7 Da. Samples were run for 30 minutes with flow rate 1.001 mL/min. Polystyrene (PS) standards were used for calibration in our experiments.

X-ray data were collected on a Rigaku XtaLAB Synergy-*i* Kappa diffractometer equipped with a PhotonJet-*i* X-ray source operated at 50 W (50kV, 1 mA) to generate Cu Kα radiation (λ = 1.54178 Å) and a HyPix-6000HE HPC detector. Crystals were transferred from the vial and placed on a glass slide in polyisobutylene. A Zeiss Stemi 305 microscope was used to identify a suitable specimen for X-ray diffraction from a representative sample of the material. The crystal and a small amount of the oil were collected on a MiTeGen 100 micron cryoloop and transferred to the instrument where it was placed under a cold nitrogen stream (Oxford 700 series) at 100K.The sample was optically centered with the aid of a video camera to ensure that no translations were observed as the crystal was rotated through all positions. A unit cell collection was then

carried out. After it was determined that the unit cell was not present in the CCDC database a data collection strategy was calculated by *CrysAlis*^{Pro}[1]. The crystal was measured for size, morphology, and color.

The chiral polymer samples were coated with a thin gold layer by sputtering, and a NANOSCIENCE Phenom ProX scanning electron microscopy (SEM) (Phoenix, AZ, USA) was used to observe the morphological characteristics.

The CD measurements were carried out using a Jasco J-815 spectrometer equipped with a Peltier MPTC-490S temperature-controlled cell holder unit. A 200-μL chiral polymer sample at a concentration of 0.2 mg/mL in HPLC gradient methanol was placed in a 1 mm quartz cuvette on the temperature-controlled cell holder. CD spectra were collected using Jasco Spectra measurement version 2 software for a wavelength range of 190–400 nm with a data pitch of 0.1 nm using a bandwidth of 1 nm and scanning speed of 100 nm/min. Each spectrum was corrected by subtraction with corresponding methanol background.

2. Synthetic Procedures

1,8-dibromo-2,7-diethoxynaphthalene

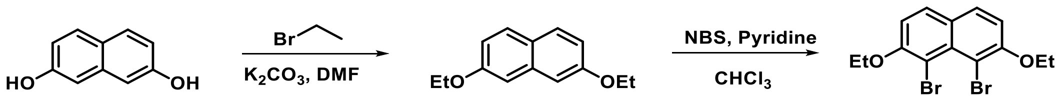

2,7-dihydroxynaphthalene (1.6 g, 10 mmol) and potassium carbonate (5.5 g, 40 mmol) were placed in a 250 mL three-neck flask, which was equipped with a stirring bar, rubber septum, and three-way stopcock. After the reaction vessel was evacuated and charged with Argon gas three times, 40 mL DMF and bromoethane (3.26 mL, 44 mmol) were added into the flask. The suspension was stirred at 70 °C for 24 h. After cooling it to room temperature, the resulting mixture was diluted with water, and then extracted three times with EtOAc. The combined organic layer was washed with brine, dried over Na_2SO_4, and then evaporated under reduced pressure. The residue was recrystallized from CH_2Cl_2-hexane to give **2,7-diethoxynaphthalene** (1.94 g, 90%) as colorless crystals for next step.
A chloroform solution of NBS (1.89 g, 10.6 mmol) in 50 mL chloroform was treated with pyridine (0.910 mL, 10.6 mmol) and the resulting solution was refluxed for 1 h. 2,7-diethoxynaphthalene (575 mg, 2.66 mmol) in 2 mL chloroform was added dropwise to the resulting orange solution over 10 min. The mixture was refluxed overnight, cooled and concentrated under reduced pressure. Column chromatography (95:5 = PE:EtOAc) yielded the **1,8-dibromo-2,7-diethoxynaphthalene** (0.826 g, 83%) as a white solid.

^{1}H NMR (400 MHz, $CDCl_3$) δ 7.68 (dd, *J* = 9.3, 6.2 Hz, 2H), 7.11 (dd, *J* = 10.0, 6.2 Hz, 2H), 4.27 – 4.18 (m, 4H), 1.54 – 1.48 (m, 6H). ^{13}C NMR (100 MHz, DMSO-d_6) (*δ*, ppm): 156.17, 131.33, 131.08, 127.39, 113.66, 105.03, 65.88, 15.37. HRMS (ESI-TOF) *m/z* calc. for $[C_{14}H_{14}Br_2O_2]^+$ 371.9361, found 371.9371.

4,7-bis(8-(4,4,5,5-tetramethyl-1,3,2-dioxaborolan-2-yl)naphthalen-1-yl)benzo[c] [1,2,5]thiadiazole(1) and 4,7-bis(8-(4,4,5,5-tetramethyl-1,3,2-dioxaborolan-2-yl) naphthalen-1-yl) benzo [c][1,2,5] selenadiazole (2)

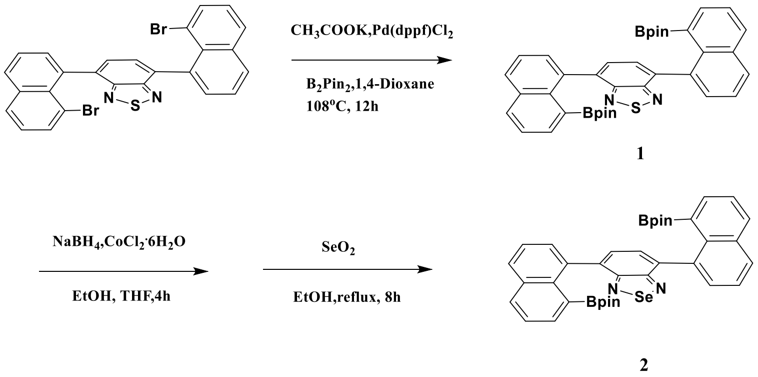

KOAc (588 mg, 6.0 mmol), 4,7-bis(8-bromonaphthalen-1-yl)benzo[c][1,2,5] thiadiazole (294 mg 1.0 mmol), bis(pinacolato)diboron (559 mg, 2.2 mmol), Pd(dppf)Cl_2 (73 mg, 0.10 mmol) and 20 mL 1,4-dioxane were added to a 50 mL round flask, equipped with a condenser. The flask was degassed under vacuum and backfilled with argon 4 times. Then the mixture was stirred at 108 ^oC for 12 hours and monitored by TLC. The reaction mixture was cooled to room temperature and the residue was purified by column chromatography using Hexane/EtOAc (10/1) as an eluent to furnish pure product as yellow solid **1**(0.413 g, 65%).

^1H NMR (400 MHz, $CDCl_3$) δ 7.96 (dd, *J* = 8.2, 1.3 Hz, 2H), 7.91 (dd, *J* = 8.1, 1.1 Hz, 2H), 7.80 (dd, *J* = 6.8, 1.3 Hz, 2H), 7.76 (dd, *J* = 7.2, 1.2 Hz, 2H), 7.59 – 7.54 (m, 4H), 7.48 (dd, *J* = 8.2, 6.8 Hz, 2H), 0.79 (d, *J* = 9.2 Hz, 12H), 0.62 (s, 12H).^{13}C NMR (100 MHz, $CDCl_3$) (δ, ppm): 155.60, 136.64, 135.82, 134.83, 134.78, 134.40, 131.08,130.24, 129.83, 129.47, 125.22, 83.59, 83.14, 25.11, 24.18.HRMS (ESI- TOF) *m*/*z* $[C_{38}H_{38}B_2N_2O_4S + H]^+$ calcd for 641.2817, found 641.2847.

Diboron derivative **1** (640 mg,1.0 mmol), $CoCl_2·6H_2O$ (24 mg, 0.1 mmol), and $NaBH_4$ (152 mg, 4.0 mmol) were added to an ethanol/THF (10 mL/10 mL) solvent mixture. The resultant reaction mixture was stirred at room temperature for around 4 hours and monitored by TLC analysis. After compound **1** was consumed completely, the solvent was removed by a rotavapor. The crude solid mass was extracted with 100L ethyl acetate, washed with brine, and dried over anhydrous Na_2SO_4. The crude product can be used for next step without further purification.

An ethanol solution of the former crude product and SeO_2 (222 mg, 2.0 mmol) were placed in a 50 mL round bottle flask under Ar condition. The mixture was stirred at 80 ^oC for 8 hours. After completion of the reaction, the mixture was extracted with DCM. The residue was dried by rotavapor and purified by column chromatography using Hexane/EtOAc (8/1) as an eluent to get pure product as yellow solid **2** (0.32 g, 47%).

^1H NMR (400 MHz, $CDCl_3$) δ 7.93 (ddd, *J* = 20.8, 8.2, 1.1 Hz, 4H), 7.83–7.74 (m, 4H), 7.58– 7.53 (m, 2H), 7.50–7.45 (m, 2H), 7.42 (s, 2H), 0.84 (s, 12H), 0.73–0.60 (m, 12H).^{13}C NMR (100 MHz, $CDCl_3$) (*δ*, ppm): 161.13, 137.34, 137.08, 134.98, 134.76, 134.33, 131.08, 130.43, 129.78, 129.26, 125.21,

125.02, 83.16, 25.17, 24.21.HRMS (ESI- TOF) *m/z* $[C_{38}H_{38}B_2N_2O_4Se+H]^+$ calcd for 689.2261, found 689.2296.

Typical polymerization procedure for **Polymer 1A-1C and 2A-2C**

Take Polymer **1A** as an example for synthesis.

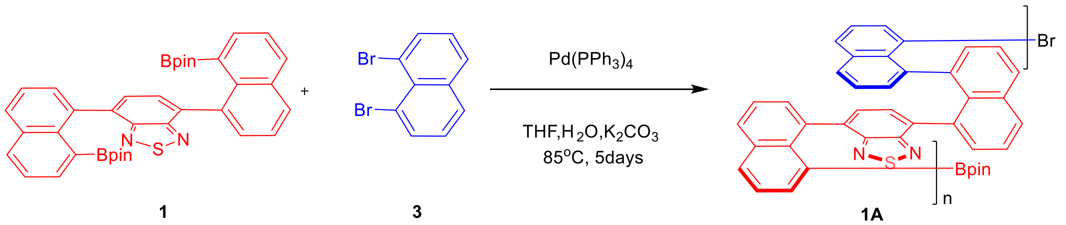

1 **3** **1A**

To an oven dried 50 mL round bottle flask under argon, **1** (128.0 mg, 0.2 mmol, 1 equiv), **3** (58.0 mg, 0.2 mmol, 1 equiv), Pd(*S*-BINAP)Cl_2 (8.0 mg, 0.01mmol, 0.05 eq) and K_2CO_3 (110.0 mg, 0.8 mmol, 4 equiv) were dissolved into 9.5 mL THF and 1.5 mL H_2O, equipped with an air condenser. The bottle flask was degassed under vacuum and backfilled with argon 6 times. It was then heated at 85 °C for 6 days. After reaction completed, the mixture was cooled to room temperature and slowly poured reaction solution into MeOH/6N HCl (25 mL/5 mL). The precipitated solution was stirred for 0.5 h at room temperature, then recovered by filtration through a Buchner funnel. The solid was separately washed with 2N HCl (10 mL) and H_2O (10 mL) for one more time, and dried at oven at 56 °C for overnight. Yellow solid (67.5 mg, 65 % yield, $[\alpha]_D^{RT}$= -7.1, c = 0.14, $CHCl_3$, MALDI-TOF_{max} (m/z = 2439). ^1H NMR (400 MHz, $CDCl_3$) δ 8.20 – 6.31 (Ar-H).

Synthesis of **Polymer 1B**

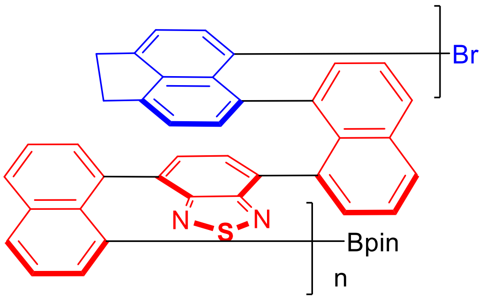

1B

Polymer **1B** was obtained as a yellow solid (54.1 mg, 49 % yield, $[\alpha]_D^{RT}$= +6.4 (c = 0.14, $CHCl_3$), Mn = 51135, Mw = 72321, Mw/Mn = 1.41). ^1H NMR (400 MHz, $CDCl_3$) δ 8.23 – 6.10 (Ar-H), 3.89 – 2.83 (CH_2CH_2-H).

Synthesis of **Polymer 1C**

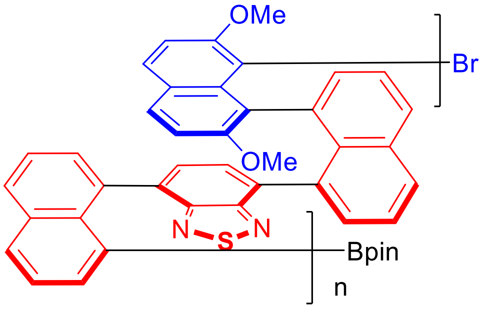

1C

Polymer **1C** was obtained as yellow solid (46.8 mg, 40% yield, $[\alpha]_{D}^{RT}$= +5.9 (c = 0.19, $CHCl_3$)) MALDI-TOF_{max} (m/z = 1533). ^{1}H NMR (400 MHz, $CDCl_3$) δ 8.19 – 6.10 (Ar-H), 4.30 – 2.95 (OCH_3-H).

Synthesis of **Polymer 2A**

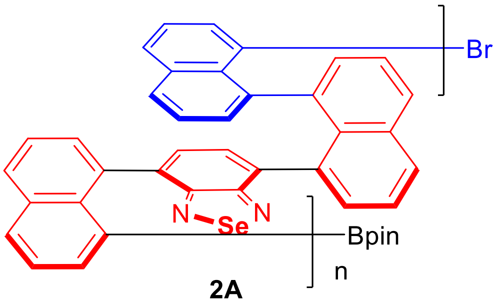

2A

Polymer **2A** was obtained as yellow solid (74.9 mg, 68 % yield, $[\alpha]_{D}^{RT}$= +4.6 (c = 0.15, $CHCl_3$), Mn = 42126, Mw = 58570, Mw/Mn = 1.39). ^{1}H NMR (400 MHz, $CDCl_3$) δ 8.08 – 6.30 (Ar-H).

Synthesis of **Polymer 2B**

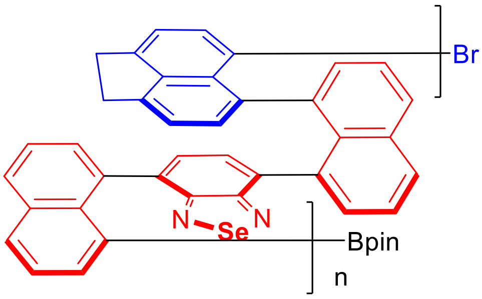

2B

Polymer **2B** was obtained as yellow solid (73.6 mg, 62 % yield, $[\alpha]_{D}^{RT}$= +11 (c = 0.08, $CHCl_3$), Mn = 45143, Mw = 55199, Mw/Mn = 1.22). ^{1}H NMR (400 MHz, $CDCl_3$) δ 8.11– 6.14 (Ar-H), 3.83 – 2.63 (CH_2CH_2-H).

Synthesis of **Polymer 2C**

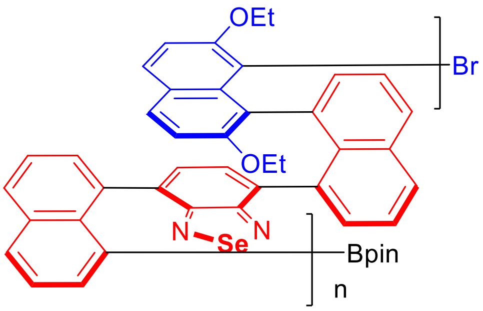

2C

Polymer **2C** was obtained as yellow solid (95.7 mg, 73% yield, $[\alpha]_{D}^{RT}$= +15 (c = 0.08, $CHCl_3$) Mn = 40854, Mw = 53136, Mw/Mn = 1.30). ^{1}H NMR (400 MHz, $CDCl_3$) δ 8.18 – 6.49 (Ar-H),3.86 – 3.00 (OEt-H(CH_2)).

3. NMR Spectra

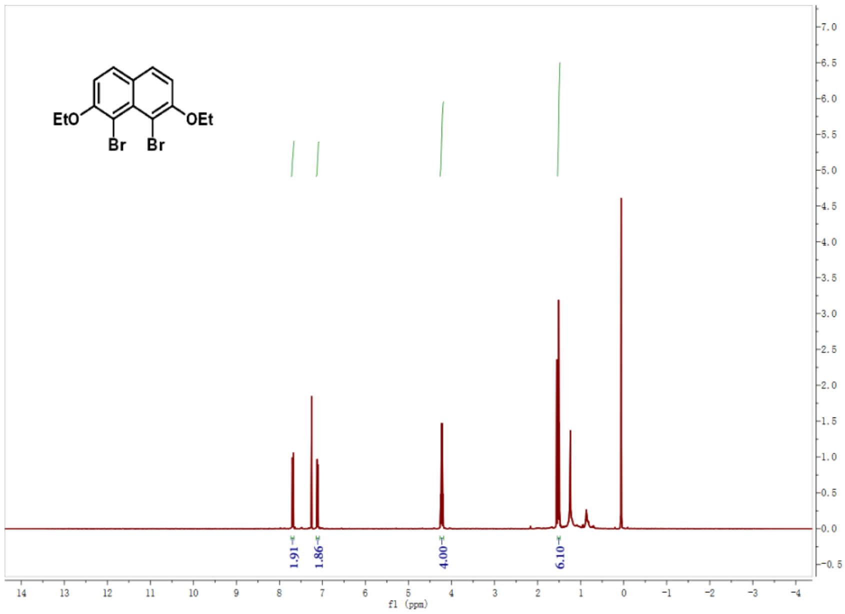

Figure S1. ^{1}H NMR spectrum of **1,8-dibromo-2,7-diethoxynaphthalene**

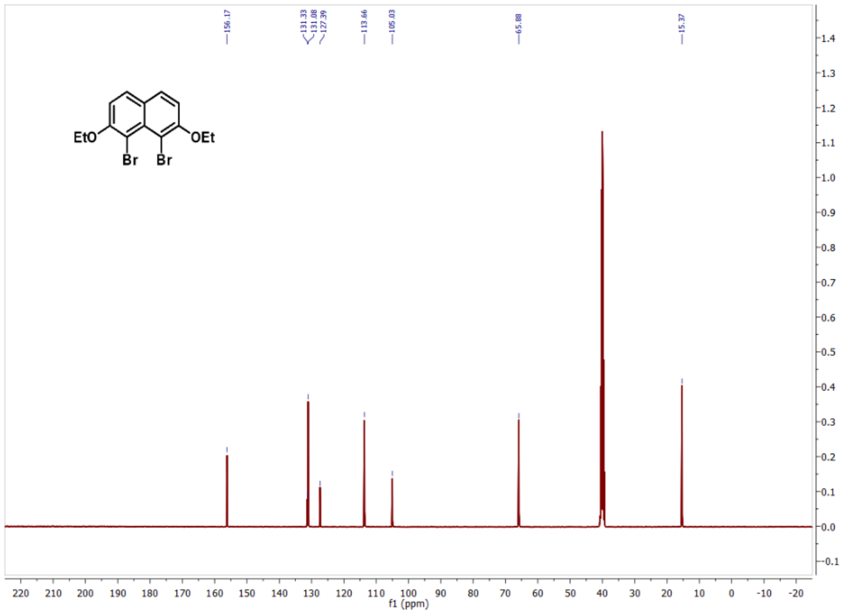

Figure S2. ^{13}C NMR spectrum of **1,8-dibromo-2,7-diethoxynaphthalene**

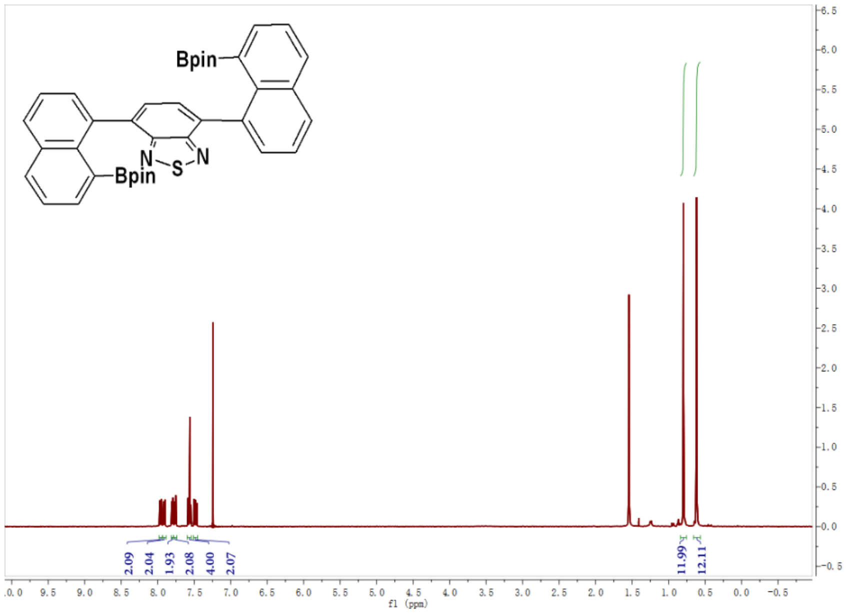

Figure S3. ^{1}H NMR spectrum of **1**

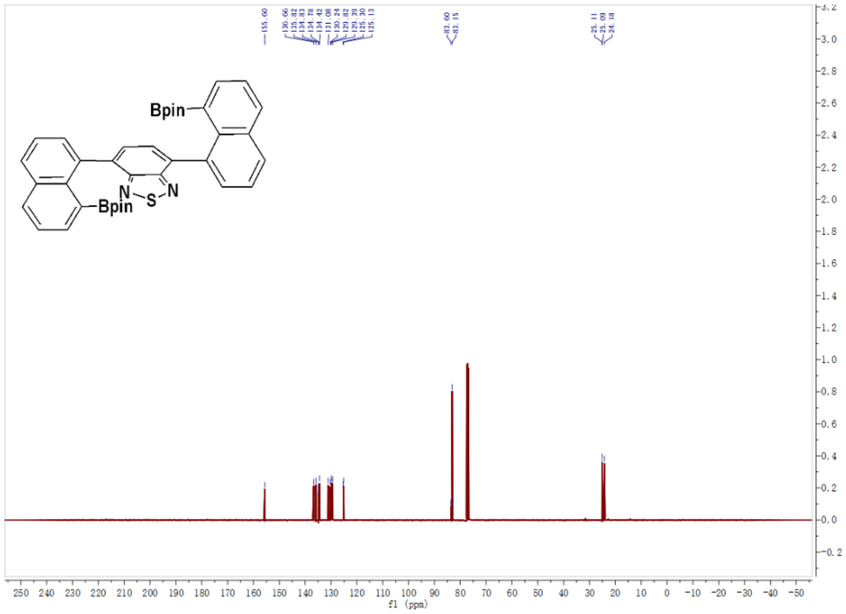

Figure S4. ^{13}C NMR spectrum of **1**

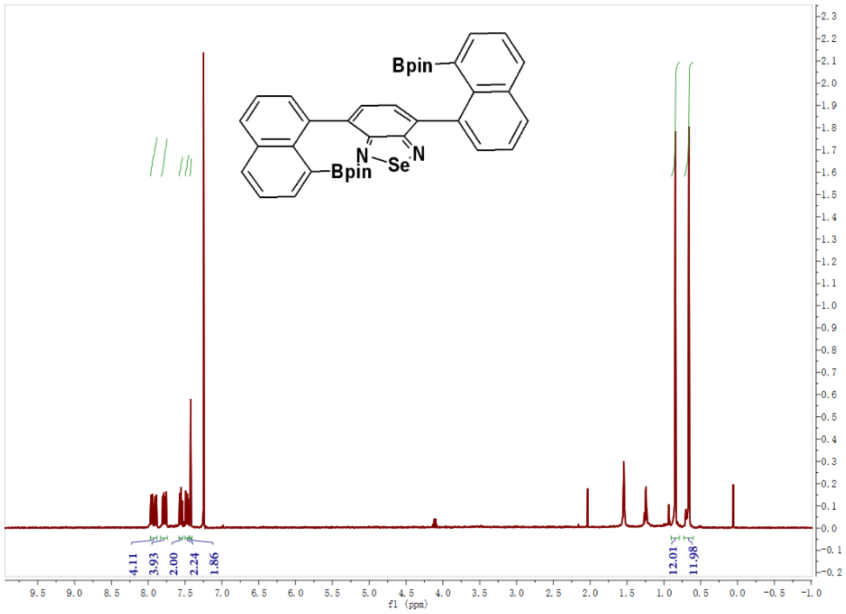

Figure S5. ^{1}H NMR spectrum of **2**

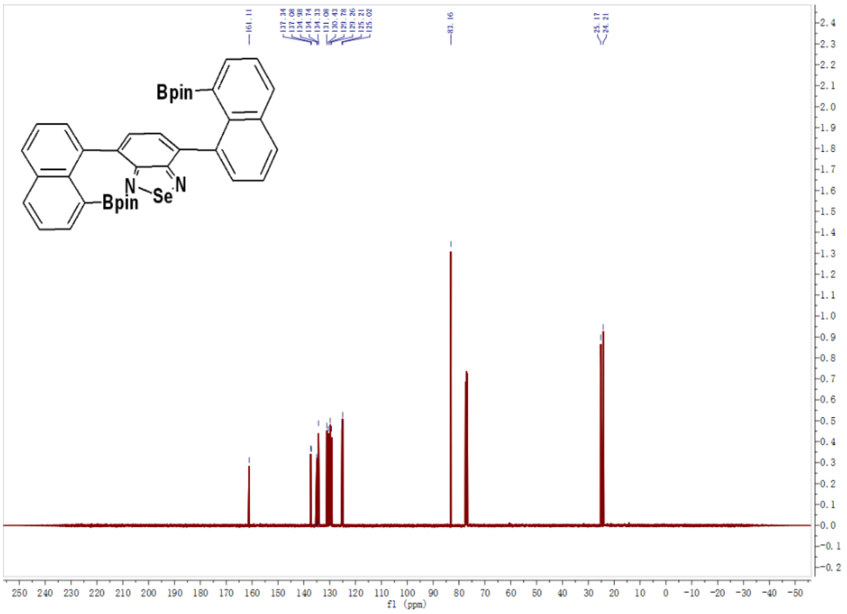

Figure S6. ^{13}C NMR spectrum of **2**

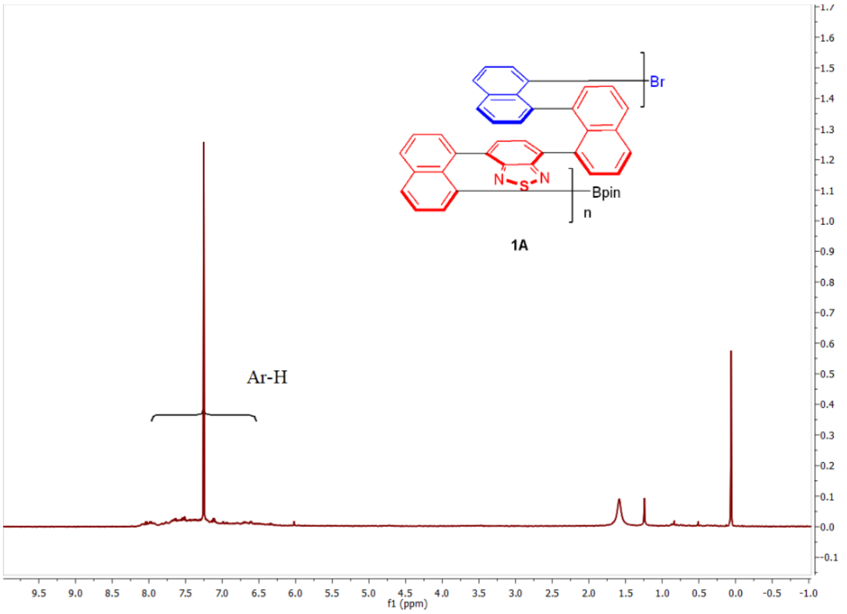

Figure S7. ^{1}H NMR spectrum of **1A**

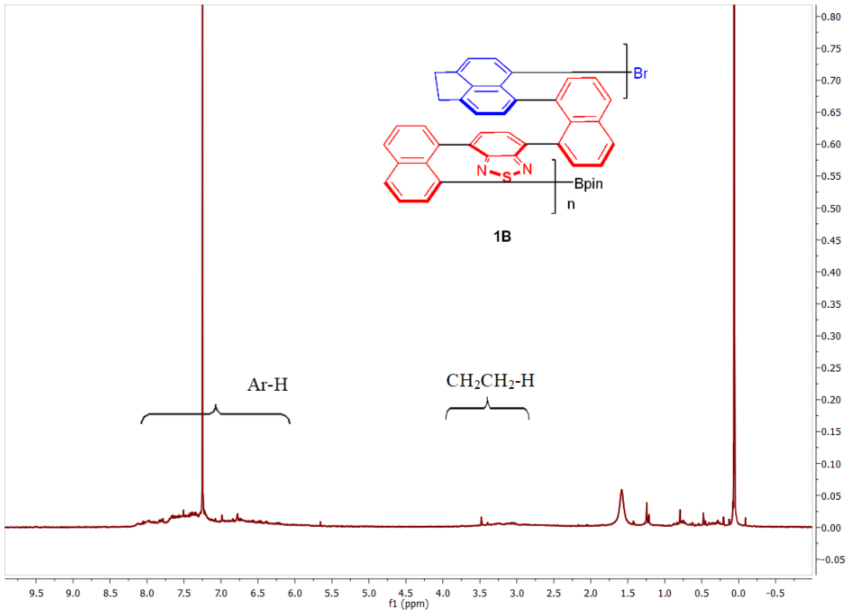

Figure S8. ^{1}H NMR spectrum of **1B**

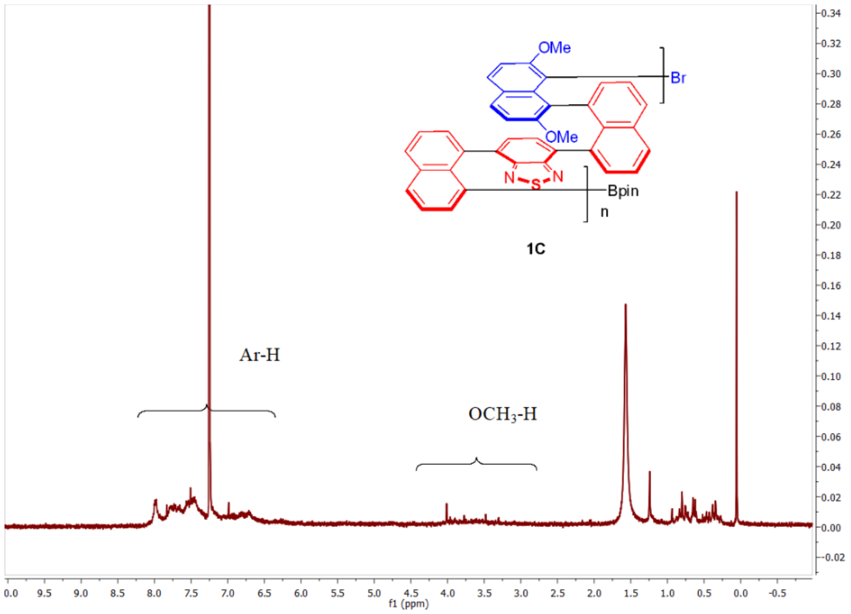

Figure S9. ^{1}H NMR spectrum of **1C**

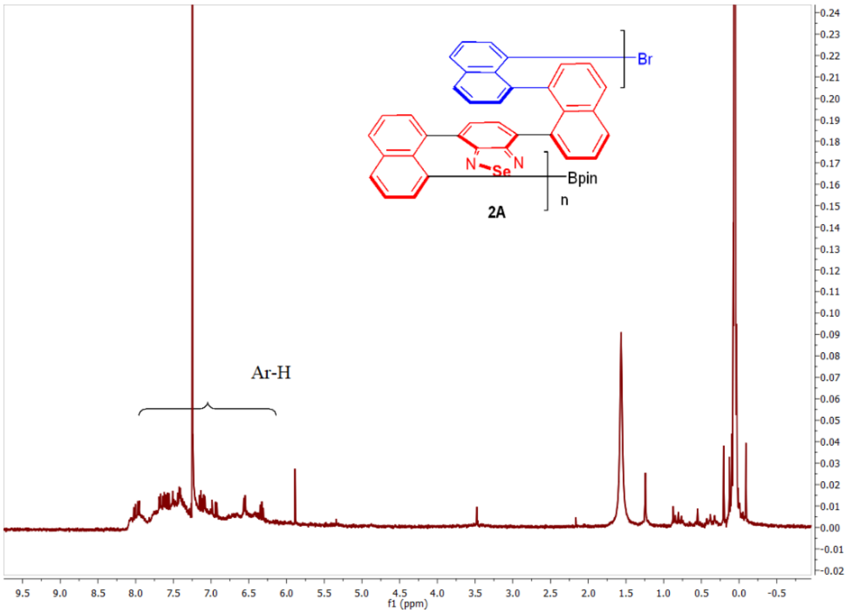

Figure S10. ^{1}H NMR spectrum of **2A**

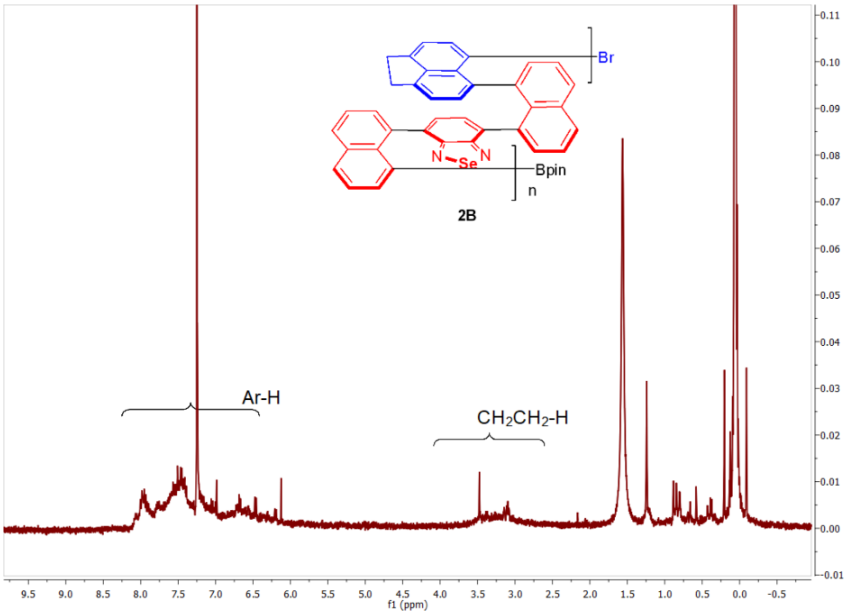

Figure S11. ^{1}H NMR spectrum of **2B**

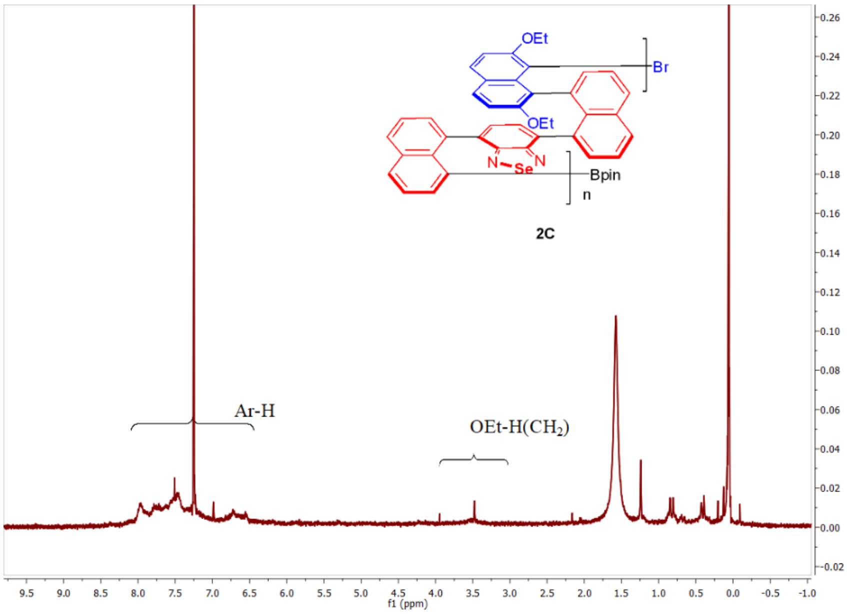

Figure S12. ^{1}H NMR spectrum of **2C**

4. GPC data

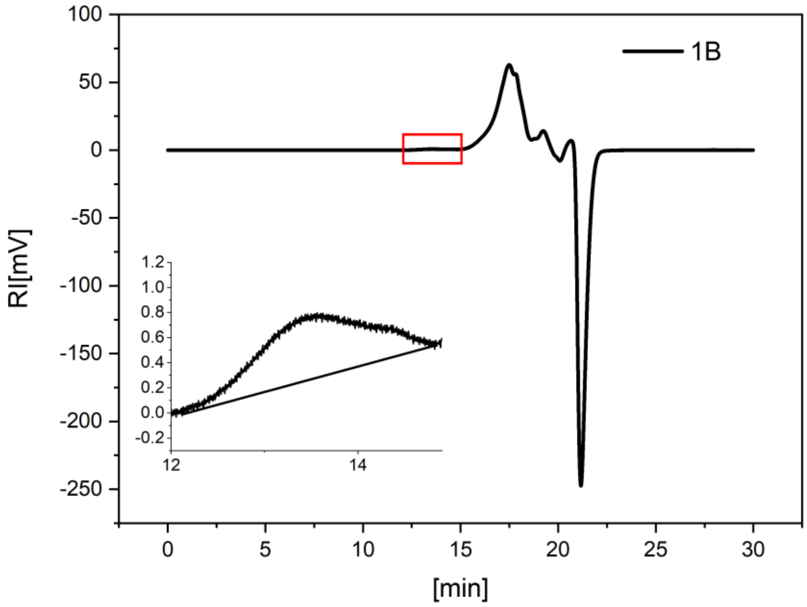

Figure **S13.** GPC data of **1B.** The inset shows the zoomed in rectangular area.

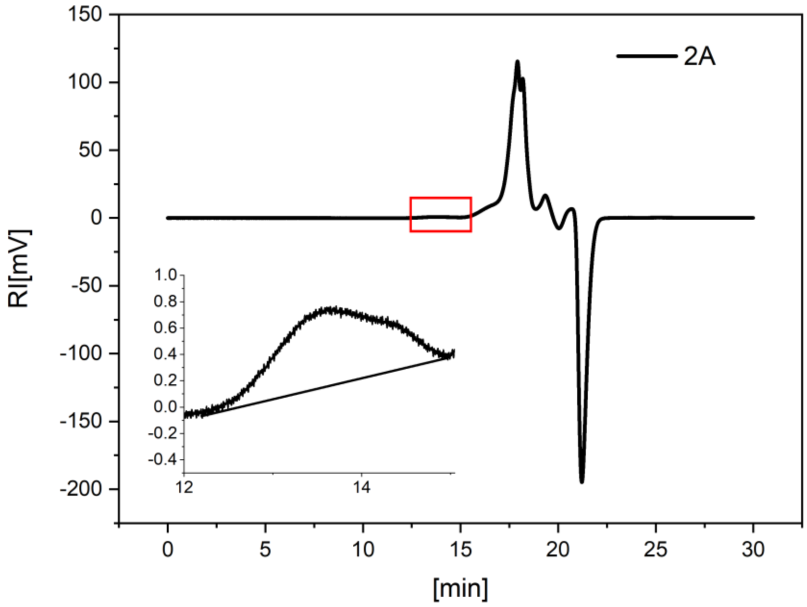

Figure **S14.** GPC data of **2A.** The inset shows the zoomed in rectangular area.

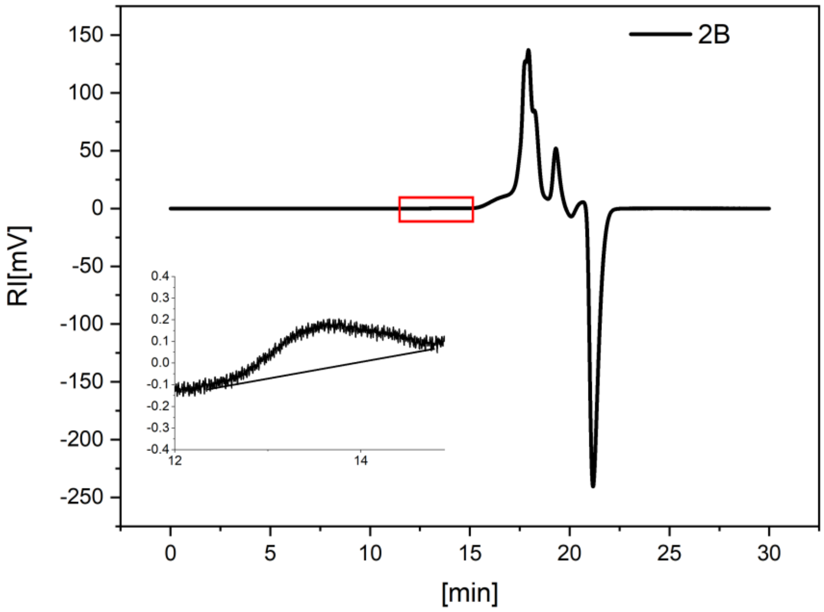

Figure **S15.** GPC data of **2B.** The inset shows the zoomed in rectangular area.

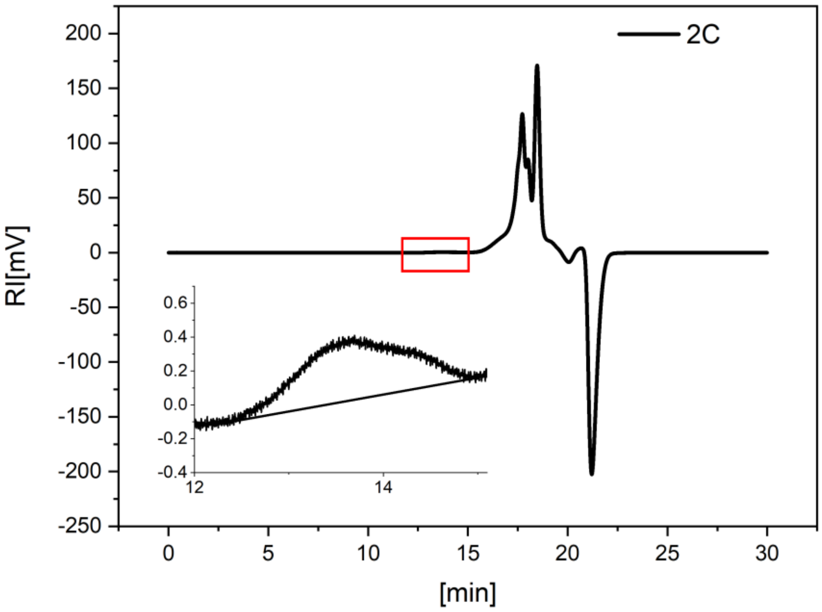

Figure **S16.** GPC data of **2C.** The inset shows the zoomed in rectangular area.

5. MALDI-TOF Analysis

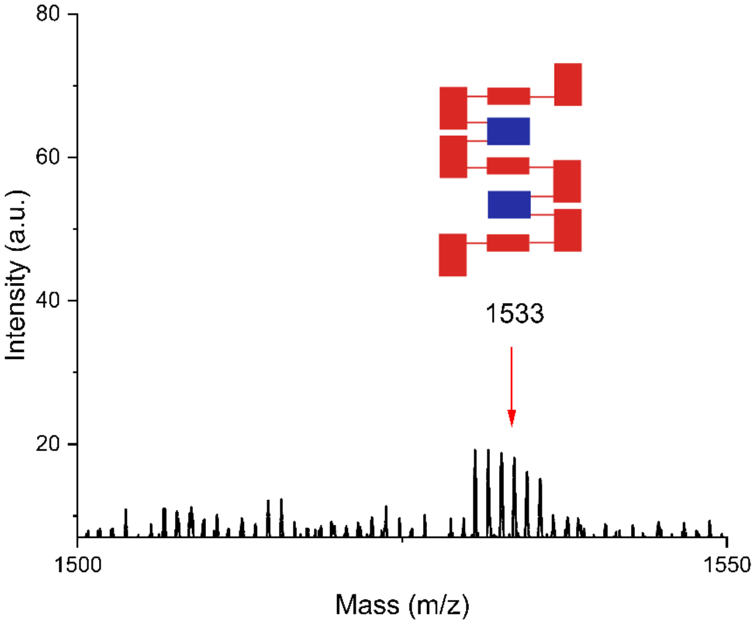

Figure **S17.** MALDI-TOF data of **1C**

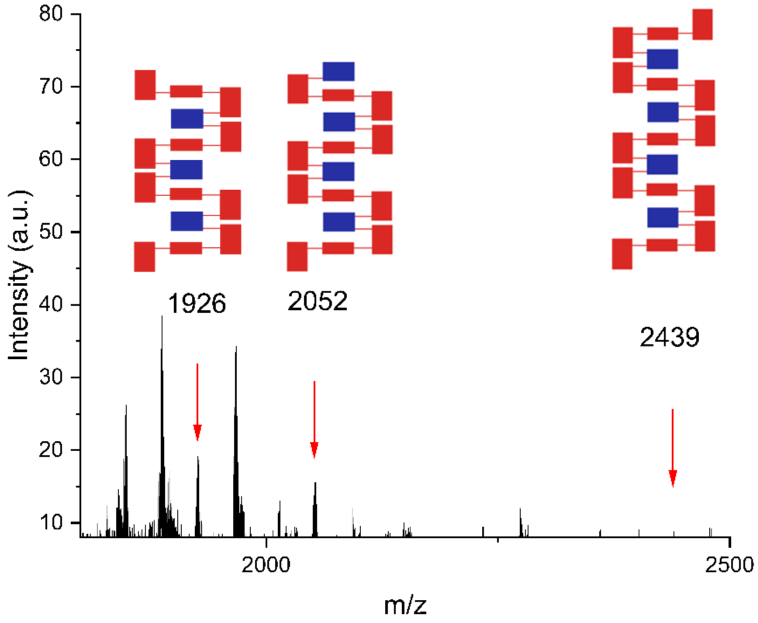

Figure **S18.** MALDI-TOF data of **1A**

6. X-ray Diffraction Information

Table S1 Crystal data and structure refinement for co-monomer

Identification code	Li20_19
Empirical formula	$C_{48}H_{31.35}N_2O_{1.18}PS$
Formula weight	717.93
Temperature/K	100.0(2)
Crystal system	triclinic
Space group	P-1
a/Å	8.95500(10)
b/Å	14.1199(3)
c/Å	15.7142(2)

α/°	67.362(2)
β/°	85.8580(10)
γ/°	74.610(2)
Volume/Å^3	1767.12(6)
Z	2
ρ_{calc}g/cm^3	1.349
μ/mm^{-1}	1.572
F(000)	748.0
Crystal size/mm^3	0.211 × 0.148 × 0.056
Radiation	Cu Kα (λ = 1.54184)
2Θ range for data collection/°	6.098 to 154.818
Index ranges	-11 ≤ h ≤ 11, -16 ≤ k ≤ 17, -19 ≤ l ≤ 19
Reflections collected	64012
Independent reflections	7266 [R_{int} = 0.0364, R_{sigma} = 0.0168]
Data/restraints/parameters	7266/642/524
Goodness-of-fit on F^2	1.066
Final R indexes [I>=2σ (I)]	R_1 = 0.0580, wR_2 = 0.1608
Final R indexes [all data]	R_1 = 0.0616, wR_2 = 0.1648
Largest diff. peak/hole / e Å^{-3}	1.23/-0.69

Refinement Details

After data collection, the unit cell was re-determined using a subset of the full data collection. Intensity data were corrected for Lorentz, polarization, and background effects using the *CrysAlis^{Pro}*[1]. A numerical absorption correction was applied based on a Gaussian integration over a multifaceted crystal and followed by a semi-empirical correction for adsorption applied using the program *SCALE3 ABSPACK*[2]. The *SHELX-2014*[3], series of programs was used for the solution and refinement of the crystal structure within OLEX2 software[4]. Within the structure a water molecule (O2) was partially occupied (0.17) and phenyl ring (C23 < C28) nearest to that site was positionally disorder in relation to the presence of the water molecule (A sites when the water molecule is present and B sites when the water molecule is absent). To help maintain reasonable ADP values and bond lengths, RIGU, SIMU and AFIX 66 restraints and constraints were applied to the disordered sites. Hydrogen atoms bound to carbon and nitrogen atoms were geometrically constrained using the appropriate AFIX commands.

7. CD spectra

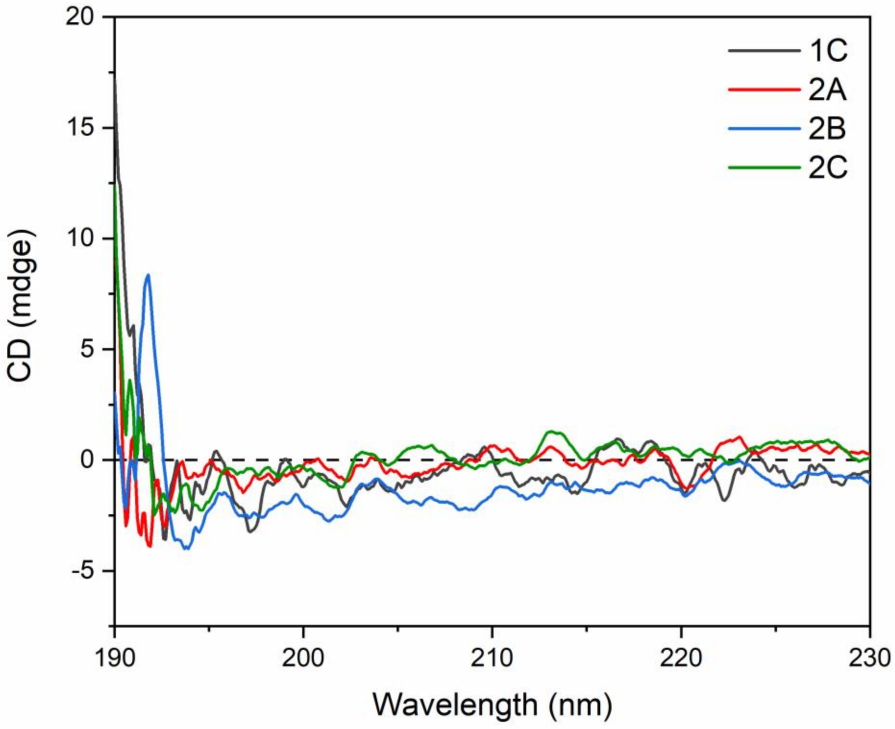

Figure **S19**. CD spectra of **1C**,**2A, 2B**, & **2C** in methanol; *c*= 0.2 mg/mL
